# Time-lagged associations between two adverse childhood experiences and later-life cognitive function through educational attainment and stroke

**DOI:** 10.1017/S135561772300036X

**Published:** 2023-07-04

**Authors:** A. Zarina Kraal, Afsara B. Zaheed, Anna Krasnova, Harita Vadari, DeAnnah R. Byrd, Laura B. Zahodne

**Affiliations:** 1 Taub Institute for Research on Alzheimer’s Disease and the Aging Brain, Vagelos College of Physicians and Surgeons, Columbia University, New York, NY, USA; 2 G. H. Sergievsky Center, Vagelos College of Physicians and Surgeons, Columbia University, New York, NY, USA; 3 Department of Neurology, Vagelos College of Physicians and Surgeons, Columbia University, New York, NY, USA; 4 Department of Psychology, College of Literature, Science, and the Arts, University of Michigan, Ann Arbor, MI, USA; 5 Semel Institute for Neuroscience and Human Behavior, University of California Los Angeles, Los Angeles, CA, USA; 6 Department of Epidemiology, Mailman School of Public Health, Columbia University, New York, NY, USA; 7 Department of General Medicine, Michigan Medicine, Ann Arbor, MI, USA; 8 Edson College of Nursing and Health Innovation, Arizona State University, Phoenix, AZ, USA

**Keywords:** cerebrovascular, cognitive aging, education, mediation, parental substance abuse, parental physical abuse, structural equation model

## Abstract

**Objective:**

Adverse childhood experiences (ACEs) have been associated with worse cognitive health in older adulthood. This study aimed to extend findings on the specificity, persistence, and pathways of associations between two ACEs and cognition by using a comprehensive neuropsychological battery and a time-lagged mediation design.

**Method:**

Participants were 3304 older adults in the Health and Retirement Study Harmonized Cognitive Assessment Protocol. Participants retrospectively reported whether they were exposed to parental substance abuse or experienced parental physical abuse before age 18. Factor scores derived from a battery of 13 neuropsychological tests indexed cognitive domains of episodic memory, executive functioning, processing speed, language, and visuospatial function. Structural equation models examined self-reported years of education and stroke as mediators, controlling for sociodemographics and childhood socioeconomic status.

**Results:**

Parental substance abuse in childhood was associated with worse later-life cognitive function across all domains, in part via pathways involving educational attainment and stroke. Parental physical abuse was associated with worse cognitive outcomes via stroke independent of education.

**Conclusions:**

This national longitudinal study in the United States provides evidence for broad and persistent indirect associations between two ACEs and cognitive aging via differential pathways involving educational attainment and stroke. Future research should examine additional ACEs and mechanisms as well as moderators of these associations to better understand points of intervention.

Adverse childhood experiences (ACEs) are potentially traumatic experiences that occur in early life. ACEs vary in frequency (e.g., problems arising from a parent’s/caregiver’s substance use versus death of a parent/caregiver) and type (e.g., physical abuse, neglect, and household dysfunction) (Krinner et al., 2021). Seminal work has shown long-term effects of ACEs on poor health outcomes in adulthood (Felitti et al., 1998), and associations have been well replicated (Petruccelli et al., 2019). Emerging work has further observed broader health effects of ACEs, including poorer cognitive performance in midlife (Richards & Wadsworth, 2004) and a higher risk of dementia in later life (Donley et al., 2018; Norton et al., 2011; Radford et al., 2017; Tani et al., 2020). These findings suggest an enduring impact of ACEs that persist over the life course into older adulthood.

## ACEs and later-life cognitive function

Findings regarding associations between ACEs and domains of cognition in later life have been mixed. Some studies suggest a dose-dependent effect of ACEs on worse cognitive functioning in older adulthood (Halpin et al., 2021), while others have not found an association between cumulative ACEs and cognition. Instead, these studies suggest that individual ACEs may be uniquely linked to cognition in older adulthood, though patterns of associations have been inconsistent (Gold et al., 2021; Kobayashi et al., 2020; O’Shea et al., 2021; Ritchie et al., 2011).

While the specific ACEs queried in studies vary, severe parental/caregiver substance abuse (e.g., frequent substance use that resulted in family problems) and physical abuse from a parent/caregiver are two commonly assessed ACEs in longitudinal cohort aging studies. Disaggregating these two ACEs may be especially important as they have been inconsistently associated with cognition. For instance, retrospectively reported parental substance abuse was independently associated with lower memory scores, but not with language or executive function, in a population-based sample of older adults in South Africa (Kobayashi et al., 2020). In contrast, other studies have not observed an association between parental substance abuse and later-life cognition (Gold et al., 2021; O’Shea et al., 2021; Ritchie et al., 2011). Furthermore, Ritchie et al. (2011) found a *positive* association between childhood parental physical abuse and verbal fluency scores in a community-based sample of older adults in France. Interestingly, another study observed a similarly unexpected association between a different aspect of childhood adversity (i.e., food deprivation) and less cognitive decline in a sample of African American older adults in Chicago (Barnes et al., 2012). Additional research examining individual ACEs in representative populations is needed to elucidate inconsistencies in the association between unique ACEs and later-life cognitive domains. Furthermore, given qualitative differences between different ACEs (e.g., parental substance abuse versus parental physical abuse), the strength and underlying pathways of their associations with cognitive outcomes may vary. A greater understanding of the mechanisms linking individual ACEs to cognitive aging outcomes may shed light on factors that confer risk for or resilience against poor cognition in later life.

## Educational and cerebrovascular pathways linking ACEs to cognitive function

One pathway that may link childhood adversity to later-life cognitive outcomes is educational attainment. Exposure to ACEs may induce stress-related physiological perturbations that engender long-lasting alterations in brain structure and function (Bick & Nelson, 2016; Hart & Rubia, 2012; Teicher et al., 2003). Neurological changes in early-life disrupt cognitive functions, including attention, executive functions, and memory, impeding intellectual development (Pechtel & Pizzagalli, 2011) and obstructing children’s academic engagement and ultimate educational attainment. In turn, low educational attainment has been linked to poor cognitive health outcomes in older adulthood (Glymour et al., 2008; Karp et al., 2004; Langa et al., 2017), in part through downstream consequences of lower socioeconomic resources (Lövdén et al., 2020; Lyu & Burr, 2016; Wickrama et al., 2013) and cognitive reserve (Stern, 2009). While few studies have utilized a mediation framework, recent studies showed that education mediated the association between retrospectively reported ACEs and concurrent cognition (Halpin et al., 2021) as well as dementia risk (Tani et al., 2020). However, results are limited by these studies’ examination of cumulative ACEs. Additional research on the long-term cognitive effects of unique ACEs may clarify the specificity and mechanisms of associations between individual ACEs and later-life cognitive functions.

In addition to pathways involving socioeconomic resources and cognitive reserve, education may also influence later-life cognition through physical health. Indeed, evidence has causally linked lower educational attainment to many physical health outcomes (Baker et al., 2011; Lleras-Muney, 2005). One health outcome that has particularly serious implications for cognitive aging is stroke (Levine et al., 2015; Tatemichi et al., 1994). Population-based studies have prospectively linked lower educational attainment to higher stroke risk (Jackson et al., 2018; Xiuyun et al., 2020), which may reflect negative health behaviors (e.g., smoking, physical inactivity; Jackson et al., 2018), depressive symptoms (Jackson & Mishra, 2013), lower health literacy (van der Heide et al., 2013), and health care access (Williams, 1990; Zajacova & Lawrence, 2018). Thus, lower educational attainment related to childhood adversity may have implications for late-life cognitive functioning through intermediary health factors, especially those affecting brain health, such as stroke.

Stroke may also link childhood adversity to poorer later-life cognitive function independent of educational attainment. Previous studies have linked childhood abuse and parental substance abuse to perturbed inflammatory responses (Carroll et al., 2013) and poor physical health outcomes (Aguayo et al., 2022). Chronic exposure to ACEs can become biologically embedded and lead to prolonged dysregulation in hormonal, inflammatory, and cardiovascular functions that impact health throughout the life course (Felitti, 2009; Miller et al., 2011; Su et al., 2015; Wade et al., 2019). Physiological dysregulation may accumulate and contribute to cerebrovascular dysfunction, heightening stroke risk in older adulthood (Jin et al., 2010; Whelton et al., 2018), and poor cognition subsequently.

## Limitations of previous work

Prior studies examining associations between ACEs and cognitive outcomes in older adulthood are limited by their assessment of cognition [e.g., using a brief, global cognitive screener (Kobayashi et al., 2020) or testing a single cognitive domain (O’Shea et al., 2021)]. Additionally, while childhood socioeconomic status (SES) has been independently associated with ACEs (for a review, see Walsh et al., 2019) and later-life cognitive outcomes (Kaplan et al., 2001; Zeki Al Hazzouri et al., 2011), most studies do not account for childhood SES as a potential confounder of ACEs-cognition associations. Despite mounting evidence on the effects of unique ACEs on cognitive outcomes, patterns of associations between individual ACEs and cognitive domains in later life remain unclear, and potential pathways linking ACEs to cognition are underdeveloped. The current study represents a first step toward clarifying some of the multifactorial mechanisms underlying the effects of ACEs on cognitive functions in older adulthood. A greater understanding of distinct paths involving sociocultural/developmental factors (e.g., educational attainment) and physical health (e.g., stroke) that may operate both independently and sequentially may reveal targets to disrupt the broad and persistent effects of ACEs on outcomes across the life course.

## The current study

The overall goal of the current study was to extend the literature on the specificity, persistence, and mechanisms of associations between ACEs and cognition. We utilized a comprehensive neuropsychological battery and a time-lagged mediation design using a large sample of participants in the Health and Retirement Study (HRS) and its Harmonized Cognitive Assessment Protocol (HCAP). Specifically, we examined the independent associations between two available ACEs (substance abuse of a parent/caregiver and physical abuse from a parent/caregiver) and five cognitive domains (Aim 1) and quantified the independent and shared contributions of educational attainment and stroke (Aim 2). Based on previous research, first, we predicted that the two ACEs would be independently associated with worse cognitive function across domains (Aim 1). Second, we predicted that three independent pathways would at least partially mediate associations between ACEs and later-life cognition: lower educational attainment (independent of stroke), stroke (independent of educational attainment), and lower educational attainment associated with stroke (Aim 2).

## Methods

### Participants and procedure

The HRS was initiated in 1992 and comprises a cohort of adults aged 51+ in the U.S. (Sonnega & Weir, 2014). HRS sampling, design, and assessment details are online (http://hrsonline.isr.umich.edu). Briefly, participants in the HRS are interviewed every two years, either in-person or via phone. These interviews collect sociodemographic and health information. Interviews are conducted in-person with a random half of the sample at every other interview wave. Beginning in 2006, in-person interviews additionally included a leave-behind psychosocial questionnaire (in which ACEs are queried). Thus, psychosocial data are available for approximately one-half of the total HRS sample at any given study wave.

In 2016, the HRS initiated the HCAP substudy, and a subset of the sample was invited to participate in a comprehensive, in-person neuropsychological assessment that included tests across domains of episodic memory, executive functioning, processing speed, language, and visuospatial function (Weir et al., 2016; Langa et al., 2020). Among participants aged 65+, a subset (26%) was randomly selected, and those who completed the 2016 core interview and venous blood collection were invited to participate in HCAP.

The current study’s baseline was defined as either 2008 or 2010 based on the wave at which a participant was eligible to receive the leave-behind psychosocial questionnaire (that included questions on ACEs). Thus, the current study’s inclusion criteria were participation in HCAP, eligibility for the leave-behind psychosocial questionnaire in 2008 or 2010, and availability of covariate data. Participant characteristics (*n* = 3304) are provided in Table 1.

In line with established procedures for time-lagged mediation analyses (Falkenström et al., 2020; Gelfand et al., 2009), we employed nonconcurrent assessments of predictors (i.e., ACEs), mediators (i.e., educational attainment and stroke history), and outcomes (i.e., cognitive domains). Specifically, the current study used data from different waves (ACEs in 2008 or 2010; mediators in 2014). Neuropsychological test data were collected in 2016 by HCAP design. All participants provided written informed consent. All study procedures were approved by the University of Michigan Institutional Review Board. This research was conducted in accordance with the Helsinki Declaration.

### Measures

#### Cognitive outcomes

Cognition was operationalized using latent variables that corresponded to five separate cognitive domains assessed in the 2016 HCAP visit: episodic memory, executive functioning, processing speed, language, and visuospatial function (Weir et al., 2016). Cognitive outcomes were determined using Confirmatory Factor Analysis in the larger HCAP sample (*N* = 3346) and have been described (Zahodne et al., 2020).

Specifically, episodic memory was assessed using ten indicators: Logical Memory (Immediate and Delayed trials) from the Wechsler Memory Scale (WMS-IV), Word List Learning (Immediate, Delayed, and Recognition trials) from the Consortium to Establish a Registry for Alzheimer’s Disease (CERAD), CERAD Constructional Praxis (Delayed trial), the Brave Man story task (Immediate and Delayed Recall Trials), and delayed word recall from the Mini-Mental State Exam (MMSE). Executive functioning was assessed via three indicators: Raven’s Standard Progressive Matrices, Number Series, and Trail-Making Test Part B (seconds). Processing speed was assessed using four indicators: Trail-Making Test Part A (seconds), Symbol Digit Modalities Test, Backwards Counting, and Letter Cancellation. Language was assessed using four indicators: animal fluency, the sum of two dichotomous items assessing visual confrontation naming from the MMSE, the sum of two dichotomous items assessing naming to verbal description from the Telephone Interview for Cognitive Status (TICS), and a single dichotomous item assessing sentence writing from the MMSE. Visuospatial function was assessed using two indicators: polygons from the MMSE and CERAD Constructional Praxis (copy).

#### Exposures

ACEs were assessed in the leave-behind psychosocial questionnaire administered in 2008 or 2010. Participants responded (yes/no) to the following questions: “Before you were 18 years old, did either of your parents drink or use drugs so often that it caused problems in the family?” (hereafter: parental substance abuse), and “Before you were 18 years old, were you ever physically abused by either of your parents?” Previous work has shown adequate test-retest reliability (kappa coefficients: 0.55–0.75) of retrospective reports on childhood experiences of physical abuse and parental substance abuse (Dube et al., 2004).

#### Mediators

Education (0–17 years) was participants’ self-reported education. Self-reported prevalent stroke (yes/no) was obtained from the 2014 HRS wave in order to have time lags between the exposures (2008/2010) and outcomes (2016).

#### Covariates

Age (birth year) was a continuous variable. Self-reported sex was a dichotomous variable. Self-reported race and ethnicity were dummy-coded into mutually exclusive groups (Hispanic (any race), non-Hispanic Black, and non-Hispanic “other”), with the largest group (non-Hispanic white) as the reference. Childhood socioeconomic status was operationalized as parental education (in years) of participants’ mother or father, whichever was higher (Haas et al., 2012). Sensitivity analyses adjusted for baseline study year (2008/2010), which was a dichotomous variable (reference:2008).

#### Statistical analysis

Analyses were conducted in Mplus, version 8. Structural equation models (SEM) were estimated using weighted least squares. Model fit was evaluated using comparative fit index (CFI), root-mean-square error of approximation (RMSEA), and weighted root mean square residual (WRMR). CFI > 0.90, RMSEA < 0.06, and WRMR > 1.0 were used to determine adequate model fit (Hu & Bentler, 1999).

**Figure 1.**
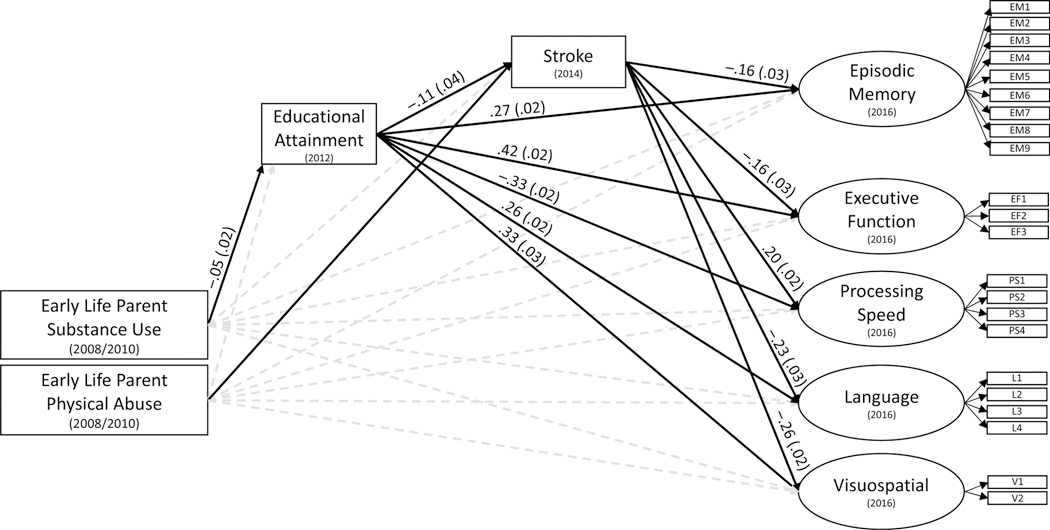
Schematic of the mediation model (Aim 2). Solid lines indicate significant (*p* < 0.05) paths, and dashed lines indicate non-significant paths. Values shown are standardized estimates with standard errors in parentheses. For processing speed, higher scores reflect worse performance (greater time to complete tasks). For all other cognitive domains, higher scores reflect better performance. For simplicity, covariates are not depicted.

For Aim 1, a single SEM tested the independent associations between the two ACEs and five cognitive domains. Cognitive domains were regressed onto ACEs and covariates, and ACEs were regressed onto covariates. For Aim 2 (mediation model), a single SEM was conducted. Educational attainment and stroke were added to the model to quantify the direct and indirect effects of ACEs on cognitive domains through three separate paths (i.e., education, stroke, and education to stroke), controlling for covariates (Figure 1). In the longitudinal time-lagged mediation model to test Aim 2, the cognitive domains were regressed onto mediators (education and stroke), ACEs, and covariates. ACEs were regressed onto covariates. In addition, stroke was regressed onto education, ACEs, and covariates. Education was regressed onto ACEs and covariates. Thus, this model included three distinct mediation paths (educational attainment, stroke, and a multi-mediator path of educational attainment and stroke). In the mediation model, “indirect effects” reflect the product of all regression coefficients within a given pathway from ACEs to a cognitive domain, independent of covariates. “Direct effects” reflect associations between ACEs and a cognitive domain, independent of mediators and covariates.

## Results

As shown in Table 1, of the 3304 participants, approximately 16.2% retrospectively reported having a history of parental substance abuse, and 7% reported having a history of parental physical abuse. The model for Aim 1 fit well (CFI = 0.954, RMSEA = 0.039, [90% CI: 0.037, 0.040], WRMR = 1.654). There were no statistically significant associations between parental substance abuse or parental physical abuse and cognitive function across domains.

The mediation model for Aim 2 fit well (CFI = 0.953, RMSEA = 0.038, [90% CI: 0.037, 0.040], WRMR = 1.627). Standardized estimates of indirect and direct effects of the two ACEs on the five cognitive domains are provided in Table 2. Standardized estimates of each statistically significant path within the mediation model are depicted in Figure 1.

### Indirect effects through educational attainment

As shown in Table 2, there were independent indirect effects of parental substance abuse on each of the five domains through a single-mediator pathway (educational attainment) and a multiple-mediator pathway (educational attainment and stroke). For the single-mediator pathway through educational attainment (solid lines in Figure 1), retrospectively reported early-life parental substance abuse that caused family problems was significantly associated with lower educational attainment (β=−0.05, SE = 0.02), which in turn was significantly associated with lower performance in each of the five cognitive domains (β=−0.33, SE = 0.02 for processing speed; βs = 0.26–0.42, SEs = 0.02–0.03 for the remaining four domains). For the multiple-mediator pathway, as noted above, parental substance abuse in early-life was significantly associated with lower educational attainment. In turn, lower educational attainment was significantly associated with a higher prevalence of stroke (β= –0.11, SE = 0.04), which in turn was significantly associated with lower cognitive performance in each of the five cognitive domains (β= 0.20, SE = 0.02 for processing speed; βs =−0.26 to −0.16, SEs = 0.02–0.03 for the remaining four domains; Figure 1). Accounting for indirect effects, there were no direct effects of parental substance abuse on cognition. There were no indirect effects of parental physical abuse on cognition through educational attainment. After accounting for indirect effects, there were no direct effects of early-life parental physical abuse on any cognitive domain (Table 2; Figure 1).

### Indirect effects through stroke

Additionally, there were unique indirect effects of early-life parental physical abuse on each of the five cognitive domains through stroke (Table 2), independent of parental substance abuse, educational attainment, and covariates. As depicted in Figure 1, parental physical abuse was associated with a higher risk of stroke [β= 0.07 (SE = 0.03)]. In turn, a history of stroke was associated with worse cognitive scores across the five cognitive domains (β= 0.20 (SE = 0.02) for processing speed; βs =−0.26 to −0.16 (SEs = 0.02–0.03) for the remaining four domains). There were no effects of parental substance abuse on cognition through stroke independent of education.

### Sensitivity and post hoc analyses

In a sensitivity analysis adjusting for ACEs data collection year (2008/2010), patterns of associations observed in primary models persisted. Post hoc models adjusted for depressive symptoms and alcohol consumption variables using data collected contemporaneously with cognition. Descriptive information is in Supplementary Table 1. When depressive symptoms were added to the models, results were similar to those in the primary models. Similar patterns of association were observed when alcohol use variables were examined.

## Discussion

The current observational study identifies potential pathways underlying broad and persistent longitudinal effects of specific adverse childhood experiences on multiple domains of cognitive functioning in older adulthood. When considered as independent predictors (Aim 1), neither ACE was associated with cognitive outcomes. In the mediation framework (Aim 2), we observed indirect associations between exposure to parental substance abuse in early life (before age 18) and lower cognitive scores assessed in later life (age 65+) through lower educational attainment and stroke. Separately, early-life parental physical abuse was associated with lower later-life cognitive scores, in part via stroke independent of educational attainment. The differential pathways through which unique ACEs were indirectly associated with cognition highlight the importance of examining experiences of childhood adversity as discrete contributors to cognitive aging. Notably, 1 in 8 children in the U.S. aged 17 or younger resides in a home with at least one parent with a substance use disorder (Lipari & Horn, 2017), and 1 in 7 children experience abuse annually (Daro & Dodge, 2009; Fortson et al., 2016). Thus, understanding the extent and drivers of associations between ACEs and later-life cognition may help identify potential points of intervention.

### Educational attainment pathway independent of stroke

The finding that educational attainment was a key driver of associations between early-life parental substance abuse and cognition across domains is in line with previous work and suggests that education mediates the association between ACEs and cognition (Halpin et al., 2021) and dementia (Tani et al., 2020). Previous studies have linked parental substance abuse to poorer academic performance and a lower likelihood of pursuing higher education (Berg et al., 2016; Mangiavacchi & Piccoli, 2018). Parental substance abuse can damage the parent-child relationship and negatively influence the home environment (e.g., a lack of structure and predictability of routines) (Solis et al., 2012; das Eiden et al., 2002), leading to poor academic engagement, attainment, and achievement [for a review, see Smith and Wilson (2016)]. Additionally, parental substance abuse has been associated with subsequent job loss (Merrick et al., 2017) and greater household financial strain (Harter & Harter, 2022), and indeed, lower childhood SES has been associated with lower educational attainment (Entwisle et al., 2005; Li et al., 2020). The current study’s observed effect of parental substance abuse on educational attainment was independent of childhood SES (operationalized as parental education). Thus, parental financial strain and unemployment, which may represent shorter-term aspects of childhood SES, may be in the causal pathway between ACEs and later-life cognitive outcomes and represent a potentially viable intervention point. Studies on interventions aimed at maintaining or increasing academic engagement are needed to assess their utility in mitigating the long-term effects of parental substance abuse on late-life cognitive morbidity.

The current study extends previous work by examining the mediating role of education in the context of later-life stroke. Specifically, we quantified the extent to which the educational disadvantage associated with early-life parental substance abuse was associated with worse later-life cognition through two separate pathways (i.e., through stroke and independent of stroke). The pathway from early-life parental substance abuse to worse later-life cognition through educational attainment that was independent of stroke may correspond to the “reserve capacity” hypothesis. Reserve capacity posits that education influences brain structure by promoting synaptogenesis, vascularization within the brain, and building cognitive reserve (Stern, 2002). Schools provide students with a cognitively challenging environment in which they acquire new knowledge, learn to retain and recall knowledge, and develop cognitive abilities to respond to novel and shifting tasks in diverse environments (Hayward et al., 2021). Thus, lower educational attainment reduces opportunities to build cognitive reserve, which is needed to lessen the likelihood or impact of age-related poor cognition (Le Carret et al., 2010; Stern, 2009). Indeed, educational attainment is a well-established independent predictor of later-life performance on tests of episodic memory, executive function, processing speed, language, and visuospatial function (Ardila et al., 2000; Heaton et al., 1986).

### Educational attainment pathway through stroke

In addition to the single-mediator pathway from early-life parental substance abuse to worse later-life cognition through educational attainment, there was also a multiple-mediator path involving education and stroke. Previous studies have provided compelling evidence that low educational attainment heightens risk of stroke (Gill et al., 2019; Xiuyun et al., 2020), in part via poor health behaviors and cardiovascular disease and their risk factors (Loucks et al., 2011; Qureshi et al., 2003). We extend previous work on education and stroke by utilizing a life course perspective. Based on our time-lagged mediation framework, retrospectively reported adversity in childhood and adolescence may be subsequently associated with lower educational attainment and stroke at different periods in adulthood, which in turn may be associated with greater cognitive morbidity in later life.

There were no indirect effects of parental physical abuse on cognitive outcomes through educational attainment because parental physical abuse was not associated with educational attainment. This finding contradicts well-established findings on the detrimental effects of early-life physical abuse on educational outcomes (Romano et al., 2015). One potential explanation is that older adults with early-life experiences of physical abuse who also had low educational attainment were not included in HCAP. Post hoc data inspection showed that older adults who retrospectively reported physical abuse had more years of education (12.9 ± 3.0) than those who did not report early-life abuse (13.1 ± 2.5). Contrastingly, participants with and without retrospectively reported parental substance abuse had comparable education (12.7 ± 2.8 and 12.9 ± 3.0, respectively).

### Stroke pathway independent of education

In the current study, stroke mediated the association between early-life physical abuse and later-life cognitive function. Strong evidence has documented deleterious effects of childhood physical abuse on risk factors for cerebrovascular disease in midlife. One study with prospectively collected data from children with objectively determined (i.e., via court records) physical abuse and non-abused, case-matched counterparts showed that abused children had more risk factors for cardiovascular and cerebrovascular diseases in midlife (Widom et al., 2012). Similar findings have been shown in middle-aged adults who retrospectively reported childhood physical abuse (Aguayo et al., 2022; Carroll et al., 2013).

The mediating role of stroke in abuse-cognition associations in older adulthood, an age period when strokes are more prevalent, is underdeveloped. Previous research in older adults has observed associations between childhood emotional neglect and pathologically defined stroke independent of education (Wilson et al., 2012). Childhood physical abuse was not associated with stroke, though the study may have been underpowered (*n* = 192) to test independent effects of multiple adversity domains on cerebrovascular outcomes. Given the frequent co-occurrence of emotional neglect and physical abuse, future research should elucidate the potentially neurotoxic effects of physical abuse, psychological abuse, and emotional neglect on cerebrovascular diseases. Overall, the current study’s findings generally align with research showing that abusive experiences in childhood are associated with worse cerebrovascular health in later life. While not designed to test causal effects, we extend prior work by suggesting that cerebrovascular pathways involving stroke may potentially link early-life physical abuse to later-life cognition.

Additional research may clarify the finding that stroke mediated the association between parental physical abuse and cognitive outcomes, but not parental substance abuse and cognition. One potential explanation may relate to different stress responses following direct physical abuse versus neglect or other negative experiences resulting from parental substance abuse. Individuals who are abused in early-life develop higher levels of distress and anxiety than non-abused children (Keyes et al., 2012; Widom, 1999) and may develop higher levels of harm avoidance, which has been associated with cerebral infarction (Wilson et al., 2014). Children who are physically abused may experience hypervigilance of their and others’ behavior as well as their home environment, which may reflect both a consequence of abuse and an (understandable) adaptation to avoid or minimize subsequent experiences of abuse. Indeed, persistent and high levels of childhood stress engender, maintain, and exacerbate chronic dysregulations in inflammatory functions that then lead to poor health outcomes across the life course (Miller et al., 2011).

Future investigations should examine whether the type of stress associated with parental physical abuse versus substance abuse may exert differential physiological effects. Research on the frequency and severity of anxiety symptoms associated with unique ACEs as well as the role of ACE-related coping behaviors on physical health throughout adulthood may clarify links between ACEs and later-life stroke. As with most large-scale studies of aging, the HRS was not designed to collect comprehensive data on early-life psychosocial experiences. Research with fine-grain measures of childhood adversity and resilience is needed to better understand the potentially broad reach of ACEs over time.

### Associations between ACEs and cognitive outcomes independent of mediators

Contrary to our hypothesis (Aim 1), we did not observe significant independent associations between parental substance abuse or parental physical abuse and cognitive outcomes independent of mediators. While our *a priori* hypotheses justified mediation analyses (Agler & de Boeck, 2017), the null total effects of ACEs on cognition were unexpected. Nonetheless, they are in line with some previous work (Gold et al., 2021; O’Shea et al., 2021), but not others. For example, O’Shea et al. (2021) observed that retrospectively reported parental substance abuse and physical abuse were not associated with memory in older adults, with similar findings by Gold et al (2021) in a separate sample of older adults. Kobayashi et al. (2020) documented an association between parental substance abuse and worse memory in older adults, though there were no associations with executive function, language, and visuospatial function, and parental physical abuse was not associated with any domain.

In contrast, a separate study observed a positive association between childhood physical abuse and verbal fluency scores, while severe abuse (operationalized as sexual abuse, physical abuse, and excessive physical punishment) was associated with worse visual memory (Ritchie et al., 2011). Differences in results between their study and ours may be due to diverging operationalizations of abuse. Selective survival may explain the null ACEs-cognition results in this and other studies. Many HRS-HCAP participants have outlived their life expectancy (Bastian et al., 2020), and ACEs heighten risk of premature mortality (Yu et al., 2022). There may be no observed association between ACEs and cognition among surviving older adults if individuals with ACEs who may have had poor later-life cognition differentially died before study enrollment. The current study’s finding of mediating pathways between ACEs and later-life cognition in the absence of an overall association highlights the importance of considering individual life course pathways to identify correlates of ACEs. Research using prospectively collected data are needed to evaluate potential causal pathways.

### Limitations and strengths

Limitations of the current study include the availability of a single time point of neuropsychological outcomes and two ACEs. As described above, other studies have documented the deleterious consequences of other ACEs (e.g., childhood sexual abuse, emotional neglect) and sources of early-life stress (e.g., being bullied in school) on mental, physical, and cognitive health outcomes. One implication of using just two ACEs is that we may be missing other important early life experiences, and their pathways, that could potentially be targeted for reducing cognitive morbidity. Taken together, future studies on the effects of additional ACEs on longitudinal cognition may clarify the role of early-life adversity in the rate of later-life cognitive decline.

Furthermore, future research should examine additional variables (e.g., posttraumatic stress disorder, depression, and substance use disorders). Mental health conditions may mediate associations among ACEs, educational attainment, physical health, and cognitive aging. Poor mental health stemming from ACEs may impede educational pursuits, while the prospective association between low education and stroke risk (Jackson et al., 2018; Xiuyun et al., 2020) may reflect depressive symptoms (Jackson & Mishra, 2013). Furthermore, the consequences of stroke on cognition may be due to post-stroke depression. In the current study, post hoc analyses showed that patterns of association did not vary meaningfully when adjusting for depressive symptoms and alcohol use variables assessed contemporaneously with cognition. Additional research on mental health mediators across the life course may clarify pathways between ACEs and later-life cognitive health.

Furthermore, while the current study focused specifically on stroke, a prominent marker of poor vascular and brain health, additional indicators of physical health (e.g., hypertension and cardiovascular conditions) that may occur earlier in adulthood and precede cerebrovascular dysregulation should be examined as they may represent points of intervention. The current study’s use of HRS data necessitated our focus on later-life physical and cognitive health outcomes. We note that vascular risk factors in middle age have been shown to have greater predictive power of later-life cognitive outcomes in comparison to risk factors measured in older adulthood. Due to the lack of available data, one implication of the current study is that we cannot compare the potential contributions of midlife health indicators to later-life stroke in associations between ACEs and cognition in older adulthood. It is possible that midlife physical health factors such as hypertension may affect cognition in older adulthood that are independent of and/or occur through later-life stroke. Furthermore, structural and social determinants of health (e.g., access to high-quality, affordable health care) likely play an important role in shaping the trajectory of health-related mechanisms underlying the effects of ACEs on later-life cognitive morbidity. Indeed, the current study’s interpretation of pathways linking ACEs to cognition in older adulthood is limited by its inclusion of just two (education and stroke) of many potential mediators experienced and accumulated over the life course.

Notably, given the prominent mediating role of education in observed associations and the well-documented links between education and cognitive aging (Glymour et al., 2008; Karp et al., 2004; Langa et al., 2017), future research should examine other dimensions of education beyond years of education (e.g., education quality, student-to-teacher ratios). Greater specificity regarding the components of educational experiences that benefit later-life cognition may improve intervention strategies. Another limitation of the current study is the low proportion of minoritized racial and ethnic groups (10% Hispanic, 15% Non-Hispanic Black, and 4.7% non-Hispanic “Other”) included in the HCAP. Education and health resources are racially and ethnically patterned, and these inequities contribute to the disproportionately higher cognitive morbidity in minoritized groups. Future studies must consider patterns of association in the context of race and ethnicity.

Additionally, the current study’s use of observational data precludes a causal interpretation of patterns of association. While SEM provides a powerful framework for testing associations, it is nevertheless a correlational method. Additionally, as with all statistical models, results are dependent on the variables included in them. While the current study focused on potentially mediating variables related to ACEs and later-life cognition and considered alternative mediators in sensitivity analyses, the inclusion of other variables could have revealed additional pathways. Future studies with greater statistical power are needed to test more comprehensive models and account for structural/social forces that drive health outcomes. Such studies are critical in order to characterize the complex effects of ACEs on later-life cognition.

Strengths of the current study include the use of a large, national sample followed longitudinally, the examination of three separate mediating pathways, and the availability of multiple validated neuropsychological measures that harmonize with other longitudinal studies conducted globally (Langa et al., 2020; Weir et al., 2016). These factors allowed us to extend previous research on the persistence and mechanisms of specific childhood adversity experiences on cognition in older adulthood.

## Conclusion

This national longitudinal study in the U.S. observed independent negative indirect associations between childhood exposure to two adverse experiences (parental substance abuse and parental physical abuse) and cognition in later-life. Low educational attainment and stroke represent key mediators of associations. Longitudinal research is needed to determine the extent to which investment in education and stroke prevention may mitigate harmful effects of ACEs on later-life cognition. We emphasize that there are likely many other intermediatory factors beyond education and stroke that underlie the effects of ACEs on the cognitive health of older adults. Nevertheless, findings from the current study represent an initial step in delineating the potential links between ACEs and cognitive aging and highlight the need for further examination of causal pathways to identify points of intervention.

## Supplementary Material

Suppl Table 1

**Supplementary material.** The supplementary material for this article can be found at https://doi.org/10.1017/S135561772300036X

## Figures and Tables

**Table 1.** Participant characteristics (*N* = 3304)

	*M* (*SD*) or %

Age (65–102 years)	75.8 (7.4)
Education (0–17 years)	12.9 (3.6)
Woman	60.7%
Hispanic, any race	10.3%
Non-Hispanic Black	15.0%
Non-Hispanic “other race”	4.70%
Non-Hispanic White	70.0%
Parent education (0–17 years)	10.4 (3.8)
ACEs questionnaire completion year (% in 2008)	42.4%
Stroke history (%)	10.6%
Parental substance abuse (% yes)	16.2%
Parental physical abuse (% yes)	7.0%

*Note.* ACEs = adverse childhood experiences. Continuous variables are represented by mean and standard deviation. Categorical variables are represented by frequency and expressed in percent.

**Table 2.** Standardized estimates of the mediation model (Aim 2) of ACEs on cognitive outcomes

	Indirect effect through education		Indirect effect through stroke		Indirect effect through educational attainment, stroke		Direct effect	
*ACE*/latent cognitive outcome	Estimate	SE	Estimate	SE	Estimate	SE	Estimate	SE

*Parental substance abuse*								
Episodic memory	−0.012	0.005*	0.003	0.005	−0.001	0.000*	0.013	0.019
Executive function	−0.019	0.007*	0.003	0.005	−0.001	0.000*	0.022	0.018
Processing speed	0.015	0.006*	−0.004	0.007	0.001	0.001*	0.004	0.017
Language	−0.012	0.005*	0.004	0.008	−0.001	0.001*	0.038	0.023
Visuospatial	−0.015	0.006*	0.005	0.009	−0.001	0.001*	0.008	0.024
*Physical abuse*								
Episodic memory	0.003	0.004	−0.011	0.005*	0.000	0.000	0.016	0.019
Executive function	0.004	0.006	−0.011	0.005*	0.000	0.000	−0.026	0.018
Processing speed	−0.004	0.005	0.014	0.006*	0.000	0.000	0.002	0.017
Language	0.003	0.004	−0.016	0.008*	0.000	0.000	0.013	0.023
Visuospatial	0.004	0.005	−0.018	0.009*	0.000	0.000	0.009	0.024

ACE = adverse childhood experience. SE = standard error.

* *p* < 0.05.
