# Supplementary material for: Time-lagged associations between two adverse childhood experiences and later-life cognitive function through educational attainment and stroke
Source: J Int Neuropsychol Soc. Author manuscript; Available in PMC 2026 Jun 29. (PMC13313567; doi:10.1017/S135561772300036X)
Supplement: Suppl Table 1 [file NIHMS2150870-supplement-Suppl_Table_1.docx]

Supplementary Table 1

Self-reported depressive symptoms and alcohol use among participants with and without retrospectively reported early life adverse childhood experiences

|  | Early Life Parental Substance Abuse | |  |  | Early Life Parental Physical Abuse | |  |
| --- | --- | --- | --- | --- | --- | --- | --- |
|  | Yes | No | *p* |  | Yes | No | *p* |
| ^a^Depressive Symptoms (possible score range: 0-8) | 1.6 (2.1) | 1.3 (1.8) | <0.05 |  | 1.7 (2.1) | 1.3 (1.8) | <0.05 |
| Alcohol Consumption (number of days per week alcoholic beverages is consumed) | 1.2 (2.2) | 1.1 (2.1) | NS |  | 1.1 (2.1) | 1.2 (2.1) | NS |
| Alcohol Consumption (number of alcoholic beverages consumed when drinking alcohol) | 0.6 (1.1) | 0.6 (1.0) | NS |  | 0.6 (1.1) | 0.6 (1.0) | NS |
| ^b^Binge/Excessive Alcohol Consumption (% yes) | 13.7% | 12.8% | NS |  | 13.9% | 12.8% | NS |

*Note.* ^a^Depressive symptoms over the past week were assessed with the 8-item Center for Epidemiologic Studies Depression scale (CES-D) modified to a yes/no format. ^b^Binge/excessive alcohol consumption is a calculated binary variable (yes/no) operationalized as whether a respondent consumed 3 (for men: 4) or more alcoholic beverages on 4 or more days per week. Continuous variables represented by mean and standard deviation in parentheses. Categorical variables represented by frequency and expressed in percent. Data were collected at the time of neuropsychological assessment in 2016. NS=not statistically significant.
